# The association between adolescents’ health and disparities in school career: a longitudinal cohort study

**DOI:** 10.1186/1471-2458-14-1104

**Published:** 2014-10-25

**Authors:** Ellen Uiters, Erica Maurits, Mariël Droomers, Marieke Zwaanswijk, Robert A Verheij, Fons van der Lucht

**Affiliations:** National Institute of Public Health and the Environment (RIVM), P.O. Box 1, 3720 BA Bilthoven, The Netherlands; NIVEL, Netherlands Institute for Health Services Research, P.O. Box 1568, 3500 BN Utrecht, The Netherlands; Department of Public Health, Academic Medical Centre, University of Amsterdam, P.O. Box 22660, 1100 DD Amsterdam, The Netherlands

**Keywords:** Health status, Education, Adolescents, Socioeconomic status, Health selection

## Abstract

**Background:**

Literature suggests that children’s educational achievement is associated with their health status and the socioeconomic position of their parents. Few studies have investigated this association in adolescence, while this is an important period affecting future life trajectories. Our study investigates the relationship between adolescents’ health and their subsequent school career, taking into account their parents’ socioeconomic position.

**Methods:**

Data of all Dutch adolescents who entered secondary education in 2003, according to the national education register, were linked to electronic health records from general practices and to data from the Dutch population register on a patient by patient basis. Secondary school career data of 2455 adolescents were available for several years, resulting in a longitudinal prospective cohort. School career was measured by the completion of secondary education within the research period.

**Results:**

For most health problems, adolescents’ health status at the moment of entering secondary education showed no association with the subsequent course of their school career. However, adolescents who had more frequent contact with their general practitioner for acute psychosocial problems (e.g. enuresis or overactive/hyperkinetic disorder), were less likely to complete their secondary education, also after adjustment for parental socioeconomic position. They were also less likely to complete their secondary education at the level of entry.

**Conclusions:**

Adolescents’ secondary school career is negatively affected by the presence of acute psychosocial health problems, but not by the presence of physical health problems. This underlines the importance of adequately addressing mental health problems in adolescence.

**Electronic supplementary material:**

The online version of this article (doi:10.1186/1471-2458-14-1104) contains supplementary material, which is available to authorized users.

## Background

Education is one of the key factors predicting social mobility [1, 2]. Failure to fulfil one’s educational potential may have long-term consequences for later occupational and social life and may add to socioeconomic disparities in adulthood [3, 4]. Understanding factors that negatively affect people’s educational career is important to design interventions to reduce these socioeconomic disparities. A person’s educational career is not only associated with individual variables, such as cognitive abilities and motivation, but also with his background, such as family resources and socioeconomic status [5]. Apart from these variables, the educational career of youngsters is affected by their health status [6–12]. Poor health of children is regarded as a predictor of low educational level in adulthood [13, 14]. The association between good health and positive school performance stems from the finding that healthy children attend school more frequently and pay more attention [15]. This relation between early health status and socioeconomic position during adulthood is often referred to as health-related selection. In this process, direct and indirect health-related selection can be distinguished. Direct health-related selection refers to a process in which early health status influences the social position that a person achieves in adulthood. Individuals in good health are more likely to move up the social hierarchy, whereas those in worse health are more likely to move down [16]. This process of health-related social mobility contributes to social class differences in health.

Alternatively, indirect health-related selection refers to a common denominator of both adult health status as well as adult socioeconomic position [17, 18]. For instance, health-related behaviours take shape during adolescence, which form the core of the health-related lifestyle in adulthood and also affect adult social status.

Investigating the association between health and educational achievement is complicated by the fact that a combination of explanatory mechanisms may take place [19, 20]. For instance, Chandola et al. distinguish six possible pathways linking education to health [19]. The main variables addressed in these pathways are: cognitive abilities, childhood socioeconomic circumstances, child and adolescent health, adult socioeconomic circumstances, health behaviours, and a person’s sense of control. A limiting factor in previous studies investigating the association between health and educational achievement is the fact that they are often based on self-reported health data. In the present study, we use national registration data (from the Dutch national education register, from electronic health records of general practices and from the Dutch population register, see Methods) to investigate the association between health and educational achievement. These databases enable us to focus on one possible pathway between education and health, i.e. the hypothesis that parental socioeconomic position is affecting children’s health as well as their educational career. For instance, children from low income families experience poorer health than their counterparts from high income families [21–23] and perform worse at school [24–26].

Most studies addressing the relationship between health status, parental socioeconomic position and educational career have been performed among primary school children [9]. Adolescence is an important period affecting subsequent life trajectories [3, 13]. Adolescent education and health are of substantial importance for adult development and psychosocial functioning, which, in turn, have a major impact on adults’ socioeconomic status [16]. However, only few studies have addressed the association between health status, parental socioeconomic position and educational career in adolescence. We investigate this association in the present study, focusing on the following two questions:

1. To what extent is adolescents’ health status at the start of secondary education associated with their subsequent school career?
2. To what extent is the relation between adolescents’ health status and their school career affected by the socioeconomic status of the parents?

## Methods

### Study population

In the Dutch health care system, virtually all residents are listed within a single general practice and 77% of the population has at least one contact with the general practitioner (GP) per year [27]. This provides the opportunity to perform population studies on the basis of routinely recorded electronic health record data of GPs. We used data derived from electronic health records of a sample of Dutch GPs participating in the NIVEL Primary Care Database and linked these on a patient by patient basis to the Dutch population register and the Dutch national education register. Linkage took place on the basis of 4 digit postal code, adolescents’ date of birth and sex. Statistics Netherlands functioned as a trusted third party, enabling the linkage between the datasets, while ensuring the privacy of the involved adolescents and GPs, according to Dutch law (Statistics Netherlands Act 2003). We selected all 3770 adolescents who entered secondary education in 2003 from this linked dataset. Adolescents listed in general practices which provided incomplete data regarding morbidity and prescriptions (n = 1236) and patients who died or emigrated in the period 2003–2008 (n = 27) were excluded. The same applies to patients aged ten years or younger or 14 years or older (n = 52), because they form a special group of adolescents, either because they were very young at the start of secondary education (<11 years old) or because they repeated at least two classes in primary school (>13 years old). In total, 2455 adolescents listed in 58 general practices were included in the analyses.

### Dutch national education register

In the Dutch educational system, children start secondary education after 8 years of primary education, usually at the age of 12. Generally, there are three types of secondary education:

1. pre-vocational secondary education (minimal duration of 4 years)
2. senior secondary education (minimal duration of 5 years)
3. pre-university education (minimal duration of 6 years)

The national education register contained information on individual school participation during secondary education for the school years 2005–2008 (the years refer to school years, meaning that 2005 refers to the school year that ran from September 2005 to July 2006). The following indicators of adolescents’ school career were constructed:

1. Completion of secondary education during the school years 2005–2008
2. Completion of secondary education at the entry level (or higher)
3. Completion of secondary education within the indicated time frame and at the entry level (or higher)

Level of entry is referring to the type of secondary education that adolescents start after finishing their primary education. A central element in the three indicators mentioned above is the completion of secondary education, as this is perceived as a useful measure of educational attainment, and is widely recognised as a minimum requirement for higher education and employment [7].

### GP electronic health records

Data about adolescents’ health status were derived from electronic health records of GPs participating in the NIVEL Primary Care Database in 2003 (at that time known as the Netherlands Information Network of General Practice) [28]. In 2003, the database included longitudinal data on morbidity, prescriptions and referrals of about 350,000 individuals listed in 85 general practices across the country. GP practices in moderately urbanised areas were slightly underrepresented compared to the national geographical distribution of GP practices. Group practices were slightly overrepresented compared to single handed practices. Patients listed in the practices are representative for the Dutch population with respect to age and sex.

Dutch law allows the use of electronic health records for research purposes under certain conditions. We did not consult an ethics committee, nor did we receive an official waiver, because according to Dutch legislation, we were legally not obliged to ask ethics approval. According to this legislation, neither obtaining informed consent nor approval by a medical ethics committee is obligatory for this kind of observational studies (Dutch Civil Law, Article 7:458) [29].

This study has been approved by the Steering Committee of the NIVEL Primary Care Database.

### Health status

Adolescents’ health status was based on symptoms and diagnoses recorded during GP contacts in 2003, and medication prescriptions issued in 2003 (year of entry in secondary education). It was impossible to use health data from subsequent years, since that would result in a substantial loss to follow up as a result of adolescents moving house or dropping out of general practices.

Symptoms and diagnoses were recorded by codes from the International Classification of Primary Care (ICPC), [30] which were subsequently clustered into broader categories of health problems [31, 32]. Contacts with the GP and prescriptions for the five most frequently occurring clusters of health problems were included in the analyses (Additional file 1). Contacts with the GP and prescriptions for less frequently presented health problems were combined into the category “remaining clusters”. The cluster classification does not cover all ICPC codes, particularly non-trauma acute somatic health problems are not covered. ICPC codes not included in a cluster were grouped into the category “not belonging to a cluster”. In 9% of contacts and 35% of prescriptions, no ICPC code had been recorded. These contacts and prescriptions were not further specified in a cluster.

The following indicators of adolescents’ health status were included in the analyses (for each of the clusters mentioned below, some examples of included health problems are given. For more details, see Additional file 1):

- Having had a GP contact or prescription (yes/no)
- Having had a GP contact or prescription for
- acute somatic health problems (yes/no), e.g. other localised abdominal pain or cough
- infections (yes/no), e.g. acute otitis media or acute upper respiratory infection
- acute somatic traumata (yes/no), e.g. concussion
- acute psychosocial health problems (yes/no), e.g. bedwetting/enuresis or overactive/hyperkinetic child
- chronic diseases (yes/no), e.g. diabetes mellitus or asthma
- health problems in the remaining clusters (yes/no)
- health problems not belonging to a cluster (yes/no)
- Number of GP contacts for the above mentioned clusters

### Dutch population register

The Dutch national population register made it possible to link all adolescents who had started their secondary education in 2003 to their parents or caregivers. The population register provided the following information about parents:

1. income of the adolescents’ parents (<= 25 percentile; >25 percentile)
2. the type of household (single parent household, yes/no)

In addition, the following demographic variables were included in the analyses:

1. adolescent sex
2. adolescent ethnicity (native Dutch or originating from a western country versus originating from a non-western country [33, 34].
3. level of urbanisation of the neighbourhood (dichotomised into very highly urban or highly urban versus moderately urban to rural) [34]. This variable was included because the level of urbanisation has been found to be related to school career (e.g. higher dropout rate in urban areas) as well as individual health status [35, 36].

### Analyses

For each of the three indicators of adolescents’ school career, we first performed logistic multilevel regression analyses to investigate the association with adolescents’ health status. Parental socioeconomic position was subsequently added to the model, followed by the demographic variables (sex, ethnicity, and level of urbanisation of the neighbourhood).

To investigate the potential modifying effect of parental socioeconomic status on the association between health status and school career, interaction terms between parental socioeconomic position and health status were included (only for the health problems which were significantly associated with school career). The binomial nature of the three indicators of school career and the clustering of patients within general practices was accommodated using multilevel logistic regression analyses (MLwiN; Centre for Multilevel Modelling, University of Bristol).

## Results

Demographic and socioeconomic characteristics of the study population are presented in Table 1. Table 2 shows the distribution of the health indicators. Overall, 63.8% of the adolescents had had contact with their GP or received a prescription in 2003. With regard to the number of contacts, 22.0% of the adolescents had more than two contacts with their GP in 2003. Specified in the different clusters, adolescents had most contacts or prescriptions for acute somatic symptoms (32.2%), infections (22.3%) and chronic diseases (16.8%). Adolescents least frequently contacted their GP or received a prescription for acute psychosocial health problems (4.4%).

**Table 1:** **Demographic and socioeconomic characteristics of the study population (n = 2455)**

Demographic variables		%
Sex	Male	50.4
	Female	49.6
Age (years old)*	11	57.1
	12	39.4
	13	3.5
Urbanisation level	Rural to moderately urban	63.1
	Very highly or highly urban	36.9
Ethnicity**	Native Dutch/western country	88.5
	Non-western country	11.5
**Socioeconomic variables**		
Single parent household	Yes	12.2
	No	87.8
Household income	<= 25% percentile	24.3
	>25 percentile	75.7

*Age at the start of 2003. Persons <11 years old and >13 years old were excluded (n = 52).

**Based on criteria from Statistics Netherlands [33].

**Table 2:** Indicators of health status of the study population (N = 2455) in 2003, in ICPC clusters*

	GP contact or prescription		Number of contacts with GP (% of adolescents)		
	N**	***%***	***1***	***2***	***>2***
Any contact/prescription	1566	63.8	23.7	13.4	22.0
Acute somatic health problems	790	32.2	19.5	6.7	5.3
Infections	547	22.3	14.5	4.4	2.3
Traumata	264	10.8	8.2	1.9	0.6
Acute psychosocial health problems	109	4.4	2.7	0.5	0.6
Chronic health problems	413	16.8	9.9	2.1	1.6
Remaining clusters ***	71	2.9	2.0	#	#
Not specified in clusters ****	194	7.9	5.2	1.1	0.4

*Clusters of health problems from the International Classification of Primary Care (ICPC).

**Number of persons.

***Includes family planning, pregnancy, childbirth, neoplasm, congenital handicap, side effects/consequences care, diverging outcomes, lifestyle and prevention.

****The cluster classification does not contain all ICPC codes, particularly non-trauma acute somatic health problems are not included.

#less than 10 adolescents.

Table 3 presents data regarding the school career of the study population. Most adolescents attained pre-vocational education as their highest level (54.5%), whereas 26.8% attained senior general education. Pre-university education was attained by 18.1% of the adolescents who started secondary education in 2003. A minority of adolescents (0.7%) dropped out of secondary education within the research period (these adolescents received “general year” as their highest attained level). Most adolescents (83.6%) who started their secondary education in 2003 had completed their education in 2008. For the majority of adolescents, the exit level of secondary education was at least the same as or higher than the entry level (79.1%). Moreover, most adolescents completed secondary education within the required time frame and at the entry level or higher (69.5%).

**Table 3:** **Indicators of adolescents’ school career (N = 2455)**

		%
Highest attained level	Pre-vocational secondary education	54.5
	Senior general education	26.8
	Pre-university education	18.1
	General year	0.7
Completion of secondary education	No	16.4
	Yes	83.6
Completion at entry level (or higher)	No	20.9
	Yes	79.1
Completion in required time frame and at entry level (or higher)	No	30.5
	Yes	69.5

The association between school career and health status (research question 1) is presented in Table 4, adjusted for differences in parental socioeconomic position and demographic characteristics. The intraclass correlations (ICC’s) were small (ranging from 0.018 to 0.035), suggesting that the results were not clustered within GP practices.

**Table 4:** **Associations between adolescents’ school career and health status, adjusted for demographic and socioeconomic characteristics**

		Completion of secondary school		Completion at entry level (or higher)		Completion in required time frame and at entry level (or higher)	
		OR	95% CI	OR	95% CI	OR	95% CI
Any	Contact/ prescription (yes/no)	1.03	(0.74-1.45)	1.11	(0.82-1.50)	1.23	(0.94-1.61)
Acute somatic health problems	Contact/prescription (yes/no)	0.91	(0.51-1.62)	0.90	(0.55-1.46)	0.97	(0.61-1.55)
	Number of contacts	0.94	(0.58-1.55)	0.93	(0.64-1.34)	0.81	(0.55-1.19)
Infections	Contact/prescription (yes/no)	0.91	(0.47-1.78)	1.11	(0.59-2.06)	0.94	(0.54-1.63)
	Number of contacts	0.94	(0.49-1.79)	0.85	(0.47-1.55)	0.87	(0.51-1.50)
Trauma	Contact/prescription (yes/no)	0.44	(0.10-1.97)	0.49	(0.12-2.01)	0.39	(0.11-1.37)
	Number of contacts	3.08	(0.57-16.69)	3.08	(0.64-14.84)	3.48	(0.85-14.26)
Acute psychosocial health problems	Contact/prescription (yes/no)	2.45	(0.79-7.55)	2.29	(0.82-6.38)	0.98	(0.42-2.30)
	Number of contacts	**0.33**	**(0.13-0.80)**	**0.39**	**(0.17-0.91)**	0.69	(0.29-1.63)
Chronic diseases	Contact/prescription (yes/no)	0.91	(0.53-1.57)	0.77	(0.49-1.24)	0.94	(0.61-1.45)
	Number of contacts	1.06	(0.61-1.85)	1.38	(0.87-2.18)	1.28	(0.84-1.96)
Remaining clusters *	Contact/prescription (yes/no)	0.70	(0.10-5.03)	0.72	(0.11-4.66)	1.11	(0.21-5.84)
	Number of contacts	1.43	(0.11-18.2)	0.87	(0.07-10.61)	0.94	(0.13-6.96)
Not specified in clusters **	Contact/prescription (yes/no)	0.63	(0.28-1.43)	0.65	(0.30-1.41)	0.69	(0.34-1.39)
	Number of contacts	1.66	(0.66-4.17)	1.46	(0.61-3.49)	1.24	(0.56-2.71)

OR: odds ratio; 95% CI: 95% confidence interval. Significant differences are printed in bold (p < 0.05).

*Includes family planning, pregnancy, childbirth, neoplasm, congenital handicap, side effects/consequences care, diverging outcomes, lifestyle and prevention.

**The cluster classification does not contain all codes from the International Classification of Primary Care (ICPC), particularly non-trauma acute somatic health problems are not included.

Only the number of GP contacts for acute psychosocial health problems was significantly associated with adolescents’ school career. The more contacts adolescents had with their GP for acute psychosocial health problems at the onset of their secondary education in 2003, the less likely they were to complete their secondary education within the research period. They were also less likely to complete their secondary education at the entry level. The most frequently occurring diagnoses within the cluster “acute psychosocial health problems” were bedwetting/enuresis and overactive/hyperkinetic child.

No significant interaction effects between parental socioeconomic position and the number of contacts for acute psychosocial health problems were found (research question 2).

## Discussion

Our study showed no association between GP consultations for somatic chronic diseases and adolescents’ school career. This suggests that physical health selection does not play an important role in influencing the school career of Dutch adolescents. Within the Dutch system, educational advice for secondary education is given at the end of elementary school. Health selection may already have occurred in this phase, resulting in children with health problems starting their secondary education at a lower level than their healthy peers. Nevertheless, our results revealed an association between school career and the number of GP contacts for acute psychosocial health problems, which may indicate that health-related selection does occur for adolescents with these type of health problems (cf., [12, 37]). This finding may indicate that particularly persisting psychosocial health problems, which result in multiple visits to the GP, interfere with adolescents’ school career.

The differential relation found between somatic and psychosocial indicators of health and school career is in line with previous research which concludes that health selection is not a universal phenomenon but may act differently for several indicators of health [38]. A large prospective cohort study showed that childhood psychological disorders had a far more important impact on various aspects of adult life (e.g., the ability to work, social mobility, income and marriage stability) than childhood physical health problems [39]. Although both childhood emotional and behavioural disorders are suggested to be associated with reduced upward social mobility, most studies emphasise the relative importance of behavioural disorders with respect to academic underachievement and impaired school functioning [40–44].

Though only a minority of adolescents (4.4%) consulted their GP for acute psychosocial health problems, and most contacts with GPs were for somatic health problems, the number of GP contacts for adolescent psychosocial health problems has increased in recent years [45]. This may imply that the number of adolescents at risk for health-related selection in secondary education will also increase in the coming years.

Most health problems did not show a significant association with adolescents’ school career. Taking into account parental socioeconomic position did not change this general picture. Moreover, we did not find a significant interaction between parental socioeconomic position and the number of contacts for acute psychosocial health problems. This suggests that the school career of adolescents from low as well as high income families is affected by the presence of acute psychosocial health problems.

The results of our study need to be considered in the light of the following limitations. First, our study focused on adolescents who attended regular secondary education. Adolescents with substantial health limitations (e.g. adolescents with severe visual, auditory or behavioural problems or impairments) often do not attend regular education but so-called “special education”. Since data about special education are not available in the Dutch education register, these adolescents were not included in our analyses. Future studies are needed to investigate whether our conclusion about the absence of general health-related selection can be replicated in a sample of adolescents attending secondary special education.

Secondly, our study focused on health problems presented in general practices. Health problems of adolescents who were hospitalised in 2003 but who did not consult their GP, were not included in our study. Previous research revealed that hospital admissions were related to school dropout only among adolescents who attended pre-university education. School dropout was positively related with the length of hospital stay and the frequency of admissions [46]. This is not in line with our outcomes, which may be explained by the fact that previous research focused on severe health problems (i.e. health problems treated in hospitals), whereas our study focused on health problems presented in general practice (which may be mild as well as more serious health problems). Moreover, because of a lack of power, we were not able to differentiate between school types in our analyses. Therefore, a subgroup analysis among adolescents attending pre-university education was not possible. Future studies could benefit from including hospital data as well as GP data.

Thirdly, we included adolescents’ health status only at the start of secondary education. We were therefore not able to investigate the effect of health problems occurring after the start of secondary school. However, particularly with respect to chronic conditions and mental health problems, we can assume that adolescents who had these health problems in 2003 also would have experienced more health problems in the following years.

Finally, the time required to complete secondary education depends on the level at which the adolescent starts his/her education (ranging from at least four years for pre-vocational secondary education to at least six years for pre-university education, see Methods). We were able to follow each adolescent’s school career for six years. This implies that the follow-up period was shorter in relation to the time required for the completion of senior secondary education (minimal duration of 5 years) and pre-university education (minimal duration of 6 years) than for pre-vocational secondary education (minimal duration of 4 years). Therefore, the completion of secondary education may have been more accurately assessed for adolescents following pre-vocational education than for the two other types of secondary education. In addition, we were not able to investigate a delayed qualification for respondents initially dropping out and returning to the educational system afterwards. Previous research showed that approximately 2% of the students in secondary education drop out, of whom 20% return and graduate within 5 years [47].

Our study also has some important strengths. First, most studies investigating the association between health status and educational career have been conducted among primary school children [9]. Since adolescence is an essential period affecting subsequent life trajectories, [3, 13] it is important also to study this association in adolescence.

Secondly, previous studies investigating the association between health and educational achievement have mainly used self-reported health data. To our knowledge, we were the first to link longitudinal, routinely registered health and administrative data to analyse the relation between health and school achievements. The use of data from GP medical records allowed us to assess health status based on the objective judgment of a medical doctor instead of using the more subjective perceived health status.

## Conclusion

Our study shows that acute psychosocial health problems, as presented in general practice, are negatively associated with adolescents’ school career. Though only a minority of adolescents consulted their GP for this type of health problems, the recent increase in number of GP contacts for psychosocial health problems among adolescents [45] may imply that the number of adolescents at risk for health related selection is also growing. Therefore, our study underlines the importance of attention for adolescents with acute psychosocial health problems, as these problems may negatively affect their school career, and poor school achievement may eventually undermine adult health.

Providing timely and adequate support to these adolescents may positively contribute to their school career (e.g. dropout prevention) and may subsequently enhance their possibilities for social and work participation as an adult. It may thereby provide mutual gains for the educational sector and the health sector and could serve as a basis to stimulate collaboration between both sectors.

## Electronic supplementary material

Additional file 1: **Indicators of health status, ICPC codes.** (DOC 38 KB)
