# Supplementary material for: The association between adolescents’ health and disparities in school career: a longitudinal cohort study
Source: BMC Public Health. 2014 Oct 25;14:1104. doi: 10.1186/1471-2458-14-1104 (PMC4216913; doi:10.1186/1471-2458-14-1104)
Supplement: Supplementary file 1 — Additional file 1: Indicators of health status, ICPC codes. (DOC 38 KB) [file 12889_2014_7197_MOESM1_ESM.doc]

**Additional file 1. Indicators of health status, ICPC codes**

I. ACUTE SOMATIC HEALTH PROBLEMS

| A1-10, 14, 17, 29 | K01-07, 29 | T01-08, 10-11, 29 |
| --- | --- | --- |
| B02-04, 29 | L01-20, 29 | U01-02, 04-07, 13-14, 29 |
| D01-06, 08-22, 24-25, 29 | N01-07, 16-19, 29 | X01-21, 29 |
| F01-05, 13-18, 29 | R01-09, 21-25, 29 | Y01-08, 16, 29 |
| H01-05, 13, 15, 29, 81 | S01-08, 20-24, 29 |  |

II. INFECTIONS

| A70-78 | K70,71 | T70 |
| --- | --- | --- |
| B70,71 | L70 | U70-72 , 88 |
| D70-73, 88 | N70-73 | W70-71 |
| F70, 72-73 | R70-83, 90 | X70-74, 84-84, 90, 91 |
| H70-74 | S09-11, 70-76, 84-85, 90, 95 | Y70-75 |

III. ACUTE SOMATIC TRAUMATA

| A80-82 | L72-81, 96 | U80 |
| --- | --- | --- |
| B76-77 | N79-81 | W75 |
| D79-80 | R87-88 | X82 |
| F75-79 | S12-19 | Y80 |
| H76-79 | T92 |  |

IV. ACUTE PSYCHOSOCIAL HEALTH PROBLEMS

| A13, 25-27 | K24-27 | S26-27 | Y24-27 |
| --- | --- | --- | --- |
| B25-27 | L26-27 | T26-27 | Z01-29 |
| D26-27 | N26-27 | U26-27 |  |
| F27 | P01-25, 27, 29, 74-79, 99 | W27 |  |
| H27 | R26-27 | X23, 27 |  |

V. CHRONIC DISEASES

| A12 | K74-94 | T81, 85-88, 90, 93 |
| --- | --- | --- |
| B80-82, 90 | L83-86, 88-91, 94-95, 97-98 | U95 |
| D89-98 | N86-89 | X87-88 |
| F91-95 | R91-96 |  |
| H82-86 | S86-88, 91, 97 |  |
